# PSA and PSA Kinetics Thresholds for the Presence of ^68^Ga-PSMA-11 PET/CT-Detectable Lesions in Patients with Biochemical Recurrent Prostate Cancer

**DOI:** 10.3390/cancers12020398

**Published:** 2020-02-08

**Authors:** Manuela Andrea Hoffmann, Hans-Georg Buchholz, Helmut J. Wieler, Matthias Miederer, Florian Rosar, Nicolas Fischer, Jonas Müller-Hübenthal, Ludwin Trampert, Stefanie Pektor, Mathias Schreckenberger

**Affiliations:** 1 Department of Occupational Health & Safety, Federal Ministry of Defense, 53123 Bonn, Germany; 2 Clinic of Nuclear Medicine, Johannes Gutenberg-University, 55101 Mainz, Germany; hans-georg.buchholz@unimedizin-mainz.de (H.-G.B.); matthias.miederer@unimedizin-mainz.de (M.M.); florian.rosar@uks.eu (F.R.); stefanie.pektor@unimedizin-mainz.de (S.P.); mathias.schreckenberger@unimedizin-mainz.de (M.S.); 3 Clinic of Nuclear Medicine, Bundeswehr Central Hospital, 56072 Koblenz, Germany; helmut.wieler@web.de; 4 Department of Nuclear Medicine, Saarland University Medical Center, 66421 Homburg, Germany; 5 Department of Urology, Helios Kliniken Krefeld, 47805 Krefeld, Germany; simplissimus@gmx.de; 6 Practice of Radiology and Nuclear Medicine, Praxis im KölnTriangle, 50679 Köln, Germany; jmh@praxis-im-koelntriangle.de; 7 Clinic of Nuclear Medicine, Klinikum Mutterhaus der Borromäerinnen, 54290 Trier, Germany; ludwin.trampert@mutterhaus.de

**Keywords:** ^68^Gallium-PSMA PET/CT, prostate-specific-antigen, prostate cancer, PSA kinetics thresholds, biochemical recurrence, optimal cutoff level

## Abstract

^68^Ga-PSMA-11 positron-emission tomography/computed tomography (PET/CT) is commonly used for restaging recurrent prostate cancer (PC) in European clinical practice. The goal of this study is to determine the optimum time for performing these PET/CT scans in a large cohort of patients by identifying the prostate-specific-antigen (PSA) and PSA kinetics thresholds for detecting and localizing recurrent PC. This retrospective analysis includes 581 patients with biochemical recurrence (BC) by definition. The performance of ^68^Ga-PSMA-11 PET/CT in relation to the PSA value at the scan time as well as PSA kinetics was assessed by the receiver-operating-characteristic-curve (ROC) generated by plotting sensitivity versus 1-specificity. Malignant prostatic lesions were identified in 77%. For patients that were treated with radical prostatectomy (RP) a PSA value of 1.24 ng/mL was found to be the optimal cutoff level for predicting positive and negative scans, while for patients previously treated with radiotherapy (RT) it was 5.75 ng/mL. In RP-patients with PSA value <1.24 ng/mL, 52% scans were positive, whereas patients with PSA ≥1.24 ng/mL had positive scan results in 87%. RT-patients with PSA <5.75 ng/mL had positive scans in 86% and for those with PSA ≥5.75 ng/mL 94% had positive scans. This study identifies the PSA and PSA kinetics threshold levels for the presence of ^68^Ga-PSMA-11 PET/CT-detectable PC-lesions in BC patients.

## 1. Introduction

Prostate cancer (PC) is the second most common type of malignant cancers and it accounts for 55% of global cancer incidence, together with lung, stomach, and breast cancer [1]. The global incidence of PC in 2012 is 1.1 million per year and accounts for approximately 7% of deaths in men [2]. PC incidence has increased to 95–116 per 10,000 persons and the incidence of death related to PC is 2 per 10,000 per person years since the introduction of prostate-specific-antigen (PSA)-screening [3].

Most patients with BC of PC are diagnosed at an early tumor stage with local disease. The primary treatment of choice for localized PC is radical prostatectomy (RP) or radiation therapy (RT). In recent years, several alternative treatment options have been approved, especially for the therapy of aggressive PC and/or metastatic spread [4,5]. BC occurs in about 30% of the patients treated, in spite of radical therapy [5,6]. BC is defined as a PSA is > 0.2 ng/mL after radical prostatectomy (RP) or > 2 ng/mL above the nadir following radiotherapy (RT) [7]. The shorter the time between primary treatment and BC is, the greater the risk for metastatic recurrence and clinical progression will be. In comparison, the longer the timeline, the higher the association of localized recurrence [4]. In this setting, PSA and PSA kinetics (i.e., PSA doubling time (PSAdt)—“which measures the exponential increase in serum PSA over time” [8] and PSA velocity (PSAvel)—an indication of the “absolute annual increase in serum PSA (ng/mL/year)” [8]) are valuable for the forecast of recurrent PC [4,9]. However the PSA alone does not indicate the localization of malignant lesions.

Cornford et al. shows imaging guidelines and salvage therapy protocols after primary treatment [7]. In patients with presumptive cancer after initial curative treatment, diagnostic imaging is challenging in the evaluation of patients with recurrent PC and/or distant metastases. Current standard imaging techniques for restaging include transrectal ultrasonography (TRUS), ^99m^Tc-diphosphonate bone scintigraphy, computed tomography (CT), and magnetic resonance imaging (MRI). Morphological imaging modalities, such as CT and MRI, often fail to visualize cancer lesions. Therefore, their accuracy is limited, especially for the detection of lymph node metastases (LNM). Other diagnostic methods, such as positron-emission tomography (PET) (with various tracers) or MR spectroscopy, which show metabolic activity, will be used, in order to minimize this limitation [10].

Hybrid PET/CT has been favored for several years and has been applied according to international guidelines to improve the diagnostic accuracy [8,11]. ^68^Gallium (Ga)-prostate specific membrane antigen (PSMA) is currently one of the most successful tracers for PC imaging due to its clinical specificity [12,13]. PSMA is a transmembrane protein that is overexpressed in 95% of PC cells, especially in higher grade PC, recurrent PC and metastatic disease [14]. In benign prostate tissue, it is only weakly or not expressed. PSMA provides an optimal target for the diagnosis of PC as well as the treatment of PC [15]. Different ^68^Ga-labelled PSMA inhibitors have been studied regarding their sensitivity and specificity to diagnose recurrence of PC such as HBED-CC, which is an efficient ^68^Ga-chelator [13,16]. A German group has developed Glu-NH-CO-NH-Lys(Ahx)-{^68^Ga-(N,N′-bis-[2-hydroxy-5-(carboxyethyl)benzyl]ethylen-ediamine-N,N′-diacetic acid)}(^68^Ga-HBED-CC-PSMA or ^68^Ga-PSMA-11), a small-molecule inhibitor for PSMA [6,16]. The HBED-CC ligand has a fast blood and organ clearance and little of the ligand is taken up into the liver. ^68^Ga-PSMA-11 PET/CT is a favorable diagnostic tool due to its excellent contrast-to-noise ratio and the improved detection of lesions. The HBED-CC conjugated PSMA inhibitor ^68^Ga-PSMA-11 precisely binds to PSMA-positive cells and it is more specifically internalized into PSMA expressing tumor cells and PSMA-positive metastatic lesion cells of PC [13,16,17]. In recent years, several other PSMA ligands have been developed for labelling with ^68^Ga and ^18^F. In particular, the ^18^F-labelled PSMA ligands continue to be researched [6,18,19]. To date, thousands of patients have been scanned with ^68^Ga-PSMA, while ^18^F-PSMA has only been used in a few hundred patients.

The current research is directed toward the development of more sensitive PET molecules for detection of BC at low PSA levels to allow for a personalized treatment planning at an early stage of recurrent disease. PSA kinetics has been proposed to supplement other diagnostic modalities in patient selection, especially with low PSA [9].

The goal of this study is to determine the best time for performing ^68^Ga-PSMA-11 PET/CT scans in patients with BC by calculating PSA and PSA kinetics thresholds for the detection and localization of recurrent PC.

## 2. Results

### 2.1. Clinical Characteristics

Table 1 summarizes the clinical and pathological characteristics of the 581 study patients. At the time of the scan, the median PSA levels were 2.98 ng/mL, range (0.2–2000). Gleason Score (GS, the grading system for determining the aggressiveness of PC) [8] was for GS ≤ 6 in 37 patients. 303 patients had GS 7 (7a + 7b), 104 patients had GS 8, and 139 patients GS > 8. 36% of the patients were treated with systemic androgen deprivation therapy (ADT). The primary treatment of most patients (493) was radical prostatectomy (85%). 88 patients (15%) were primarily treated with radiotherapy (Table 1).

### 2.2. Overall Positivity Rate of ^68^Ga-PSMA-11 PET/CT

^68^Ga-PSMA-11 PET/CT showed a positivity rate in 450 out of 581 patients (77%) (mean PSA 18.21 ± 101.91 ng/mL). The discrimination of the PSA values between patients with a PSMA-positive scan and those with a PSMA-negative scan was statistically significant (*p* < 0.001). The mean PSA levels of patients with positive scans were significantly higher than those of patients with negative scan results (22.94 ± 115.38 ng/mL versus 1.95 ± 3.28 ng/mL, *p* < 0.001, Mann-Whitney-U test) (Table 2).

This retrospective analysis includes 581 patients with a BC. We looked at the two groups of patients with different BC by definition (patient-group RP and patient-group RT) separately to ensure proper statistics and a homogeneous patient collective.

### 2.3. Positivity Rate of ^68^Ga-PSMA-11 PET/CT in Detecting Clinical Recurrence Post-Prostatectomy

^68^Ga-PSMA-11 PET/CT revealed malignant prostatic lesions in 370 of 493 patients (75%). A statistically significant demarcation in PSA values was shown (*p* < 0.001) between patients with a positive (3.20 ng/mL) and a negative (0.70 ng/mL) ^68^Ga-PSMA-11 PET/CT scan.

The detection efficacy of ^68^Ga-PSMA-11 PET/CT for post-prostatectomy (patient-group RP) was 40% (27) for PSA levels of 0.2 to < 0.5 ng/mL and 62% (48), 70% (61), 84% (99), 94% (135) for PSA levels of 0.5 to < 1 ng/mL, 1 to < 2 ng/mL, 2 to < 5 ng/mL, and ≥ 5 ng/mL, respectively (*p* < 0.001) (Table 3A).

Table 3A shows the sites of lesions that were detected by ^68^Ga-PSMA-11 PET/CT scans in patient-group RP. Local recurrence was evident in 29% (109/370) of the patients with a positive scan. Out of all patients with positive scans, 85% showed metastases. 54% of these patients exhibited local metastases and 23% distant metastases. Multiple metastases were observed in 66% of these patients. Lymph node (LN) metastases (LNM) were evident in 78% of the patients, 75% of which were local, 8% distant LNM, and 18% were local and distant LNM. Bone metastases were revealed in 43% of the patients with positive ^68^Ga-PSMA-11 PET/CT scans. Visceral metastases rarely occurred in 7% (Table 3A). Single metastases were detected in 28% of the patients (*n* = 105) with positive ^68^Ga-PSMA-11 PET/CT results and 56% of the patients (*n* = 208) showed multiple PC metastases (Table 4).

### 2.4. Positivity Rate of ^68^Ga-PSMA-11 PET/CT in Detecting Clinical Recurrence Post-Radiotherapy

Overall, 80 of 88 patients had a positive ^68^Ga-PSMA-11 PET/CT scan (91%). The discrimination in PSA values between the patients with a positive (7.15 ng/mL) and a negative (5.25 ng/mL) scan was not statistically significant (*p* = 0.384).

For patients that were treated with RT, the detection efficacy of ^68^Ga-PSMA-11 PET/CT was 88% (29) for PSA levels of 2 to < 5 ng/mL and 93% (51) for PSA levels of ≥ 5 ng/mL, respectively (*p* = 0.44) (Table 3B).

Table 3B shows the sites of lesions that were detected by ^68^Ga-PSMA-11 PET/CT scans in patient-group RT. In 63% (50/80) of the patients with a positive scan, local recurrence was detected. Of the positive scans, 64% showed metastases and 43% of these patients exhibited local metastases, whereas 27% showed at least one distant finding (extra pelvic LN and/or bone and/or visceral metastases). Multiple metastases were demonstrated in 75% of these patients. A total of 69% of the patients showed LNM (74% of them local, 9% distant, and 17% local and distant LNM). Bone metastases were revealed in 34% of the patients with positive ^68^Ga-PSMA-11 PET/CT scans. Visceral metastases were detected in only 4% of the positive scans (Table 3B). Multimetastatic disease was shown in 48% of the PSMA-positive patients (*n* = 38), whereas in 16% of the patients (*n* = 13) single PC metastases were detected (Table 4).

### 2.5. PSA and PSA Kinetics Threshold Levels

The performance of ^68^Ga-PSMA-11 PET/CT in relation to the PSA value at the time of the scans, as well as PSA kinetics, were assessed by receiver-operating-characteristic-curve (ROC) generated by plotting sensitivity versus 1-specificity. A PSA value of 1.24 ng/mL was found to be the optimal cutoff level for predicting positive and negative scans by means of ROC analysis (AUC = 0.788; 95% CI 0.746–0.831) in all the patients primarily treated with RP and with RT (the cohort of 581 patients).

### 2.6. PSA and PSA Kinetics Threshold Levels Post-Prostatectomy

**Figure 1:**
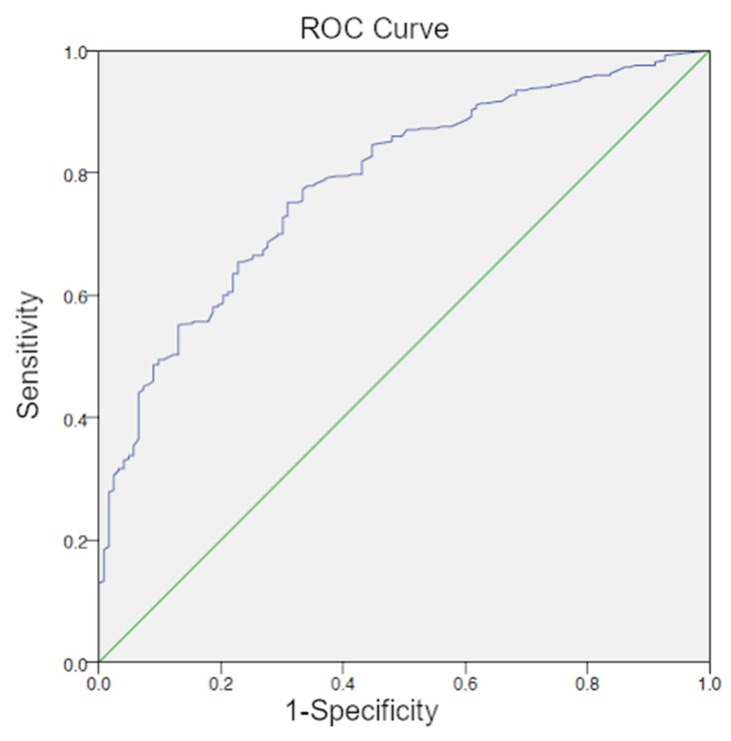
Receiver-operating-characteristic-curve (ROC) curve for PSA (patient-group RP) with an optimal cutoff level of 1.24 ng/mL.

The optimal cutoff level was also 1.24 ng/mL for patients that were treated with radical prostatectomy (370/493 patient-group RP) (AUC = 0.784; 95% CI 0.740–0.828) (Figure 1).

In patients with a PSA value <1.24 ng/mL, 52% (92/177) ^68^Ga-PSMA-11 PET/CT scans were positive, whereas patients with a PSA ≥1.24 ng/mL exhibited positive scan results in 87% (278/316) (*p* < 0.001). Local recurrence in the prostate bed was noted in 13% vs. 27% of the patients with PSA levels below and above the cutoff. Multimetastatic disease was determined in 21% of the patients with PSA values that were below cutoff level vs. 79% of patients with PSA above cutoff (*p* < 0.001, r 0.315) (Table 5A). The local and distant metastases were determined in 6% (11/177) of patients with PSA values below the cutoff level vs. 20% (62/316) of patients with PSA above cutoff (*p* < 0.001) (Table 5A). Local metastases were detected in 51 patients (29%) below and in 118 patients (37%) above cutoff PSA values (*p* < 0.001). Distant lesions were identified in 9% below vs. 18% above cutoff levels, respectively (*p* < 0.001; Table 5A). As compared to patients with PSA values that were below the optimal cutoff level, patients with PSA levels ≥ 1.24 more frequently exhibited LNM (25% vs. 75%, *p* < 0.001, r 0.228) and bone metastases (18% vs. 82%, *p* < 0.001, r 0.235).

**Figure 2:**
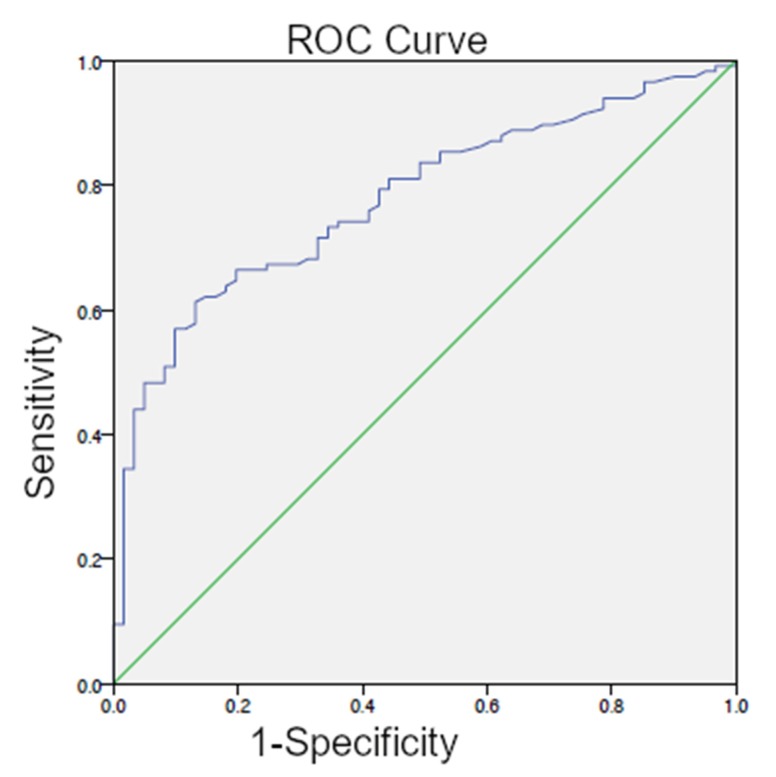
ROC curve for PSAvel (patient-group RP) with an optimal cutoff level of 1.32 ng/mL/year.

The optimal PSAvel threshold from ROC analysis for the detection of recurrent PC-lesions (patient-group RP) was 1.32 ng/mL/year (AUC = 0.777; 95% CI 0.709–0.845) (Figure 2), which showed significant differences between the PET-positive and PET-negative scans (*p* < 0.001).

The ROC curve analysis showed that PSAdt was not useful in distinguishing between patients with positive and negative ^68^Ga-PSMA-11 PET/CT scan results with an AUC of 0.450.

### 2.7. PSA and PSA Kinetics Threshold Levels Post-Radiotherapy

**Figure 3:**
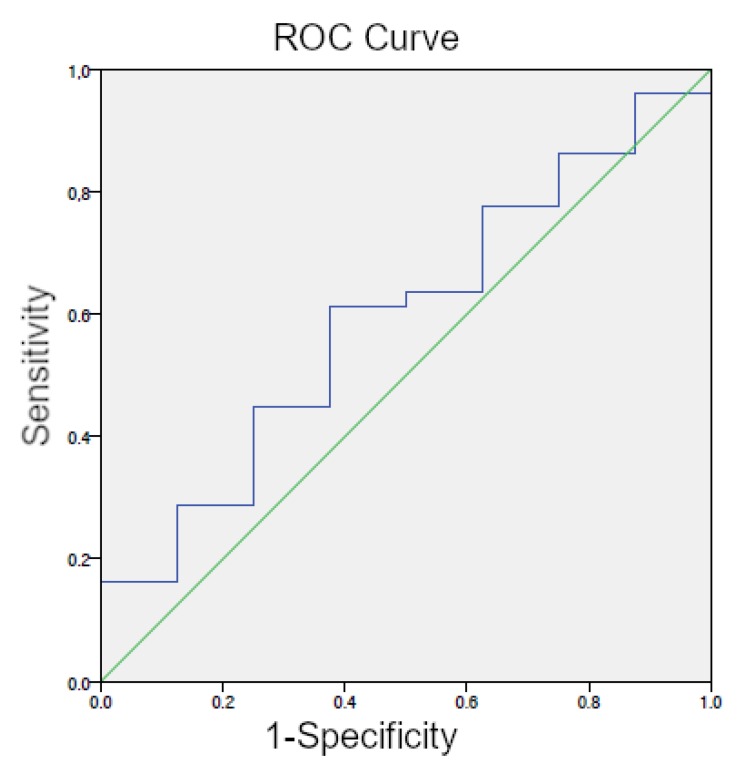
ROC curve for PSA (patient-group RT) with an optimal cutoff level of.5.75 ng/mL.

The optimal cutoff level for patients that were previously been treated with RT (80/88 patient-group RT) was 5.75 ng/mL (AUC = 0.594; 95% CI 0.400–0.788) (Figure 3). In patients with a PSA value <5.75 ng/mL, 86% (31/36) ^68^Ga-PSMA-11 PET/CT scans were positive, whereas patients with a PSA ≥5.75 ng/mL exhibited positive scan results in 94% (49/52) (*p* = 0.19, *r* 0.139). Local recurrence in the prostate appeared in 61% vs. 54% of the patients with PSA levels that were below and above cutoff, respectively. Multimetastatic disease was determined in 53% of the patients with PSA values that were below cutoff level vs. 56% of patients with PSA above cutoff (*p* = 0.008, *r* 0.255) (Table 5B). Local and distant metastases were found in 11% (4/36) of patients with PSA values below cutoff level vs. 21% (11/52) of patients with PSA above cutoff (*p* = 0.01, *r* 0.286) (Table 5B). Patients with PSA levels ≥ 5.75 more frequently exhibited LNM (33% vs. 41%, *p* = 0.393, *r* 0.076) and bone metastases (26% vs. 41%, *p* = 0.089, *r* 0.152) as compared to patients with PSA values below the optimal cutoff level.

An optimal PSAvel threshold from ROC analysis for the detection of recurrent PC-lesions (patient-group RT) could not be ascertained, since only 19 patient data sets were available for the calculation of the value, of which a positive scan result was obtained in 18 patients.

ROC curve analysis revealed a PSAdt threshold of 10.6 months (AUC = 0.600; 95% Cl 0.366–0.834) to distinguish patients with positive and negative ^68^Ga-PSMA-11 PET/CT, but without significance (*p* = 0.353).

### 2.8. Maximum Standardized Uptake Value (SUV_max_)

As evidenced by the Kolmogorov-Smirnov-Test, the SUV_max_ of the detected sites of malignant lesions were not normally distributed. SUV_max_ was the highest in bone metastases (mean and standard deviation/SD: 22.14 ± 22.98, median: 15) and lowest in lung metastases (mean and SD: 9.84 ± 9.31, median: 6). SUV_max_ of LNM and visceral metastases showed values in between.

### 2.9. GS

4% of patients with a positive ^68^Ga-PSMA-11 PET/CT scan were categorized as being low risk PC (International Society of Urological Pathology (ISUP) grade 1; GS < 7), whereas 12% of PSMA-positive patients were categorized GS 7a (ISUP grade 2; intermediate risk), 35% GS 7b (ISUP grade 3; intermediate risk), 22% GS 8 (ISUP grade 4; high risk), and 26% GS > 8 (ISUP grade 5; high risk) [8] (Table 6). High risk (GS 8, GS > 8) as compared to low risk patients (GS < 7) showed a high frequency of positive scan results (*p* < 0.001) and metastases (52% vs. 2%) (*p* < 0.001). When considering local recurrence, the results are similar, but not statistically significant. With regard to the subgroups (GS 7a vs. GS 7b), there is an important distinction between patients with intermediate risk and grade group 2 (GS 7a also called 7 (3 + 4)) (10%) when compared to patients with intermediate risk and grade group 3 (GS 7b also called 7 (4 + 3)) (36%) for PSMA-positive metastases (*p* < 0.001) (Table 6).

### 2.10. ADT

ADT was significantly associated with increased probability of a positive ^68^Ga-PSMA-11 PET/CT scan (*p* < 0.001). 37% of PSMA PET/CT-positive and 18% of PSMA PET/CT-negative patients were treated with ADT (*p* < 0.001, *r* 0.173) in the patient-group RP. For the patient-group RT, 59% of the patients with a positive PSMA scan and 63% with a negative scan were treated with androgens (*p* = 0.837, *r* = ™0.022).

### 2.11. Alkaline Phosphatase (AP)

AP has been described as an efficient and functional biomarker for the prognosis of PC (43). We wanted to verify whether AP is a predictor for bone involvement of PC. In our study, AP levels of patients with positive ^68^Ga-PSMA-11 PET/CT scan results were higher than those of patients with negative scans (93 ± 53 IU/L vs. 74 ± 24 IU/L, *p* < 0.001, Mann-Whitney-U test). Patients exhibiting bone metastases showed higher AP than patients without bone metastases (108 ± 70 IU/L vs. 76 ± 23 IU/L; *p* < 0.001).

### 2.12. Subpopulation

EAU guidelines suggest PSMA PET/CT for restaging PC in the case of patients that were treated with RP, if the PSA level is > 0.2 ng/mL and if the results would influence subsequent treatment decisions [8]. In the event that PSMA PET/CT is not available and the PSA level is ≥ 1 ng/mL, PET/CT with other tracers (Fluciclovine or Choline) has been suggested, if the results could influence subsequent treatment decisions [8]. For PSA recurrence after RT, EAU guidelines recommend performing PSMA PET/CT (if available) or fluciclovine PET/CT or choline PET/CT in patients that qualified for curative salvage treatment [8]. Therefore, we analyzed the PSA range from 0.2 ng/mL to < 1 ng/mL for post-prostatectomy patients. We did not form a separate subgroup for the irradiated patients, as no PSA threshold was stated in the EAU guidelines with regard to them.

All of the prostatectomized patients, 29% (145/493) exhibited PSA levels between 0.2 and < 1 ng/mL and 52% of these patients showed positive PET/CT scans (positive: 0.2 to < 0.5 ng/mL 40% and 0.5 to < 1 ng/mL 62%; *p* < 0.001, *r* 0.413) (Table 3A). In this subpopulation (overall positivity rate in scans and PSA levels between 0.2 ng/mL and < 1 ng/mL), ^68^Ga-PSMA-11 PET/CT showed the presence of local recurrence in the prostate bed in 27% (20 of 75), of local metastases in 59% (44 of 75), of distant metastases in 13% (10 of 75), and of local and distant metastases in 9% (7 of 75). Multimetastatic carcinoma was detected in 44% (33 of 75) (Table 3A). The absolute PSA value at the time of the ^68^Ga-PSMA-11 scan was associated with an increased probability of a positive scan result (*p* < 0.001).

## 3. Discussion

**Figure 4:**
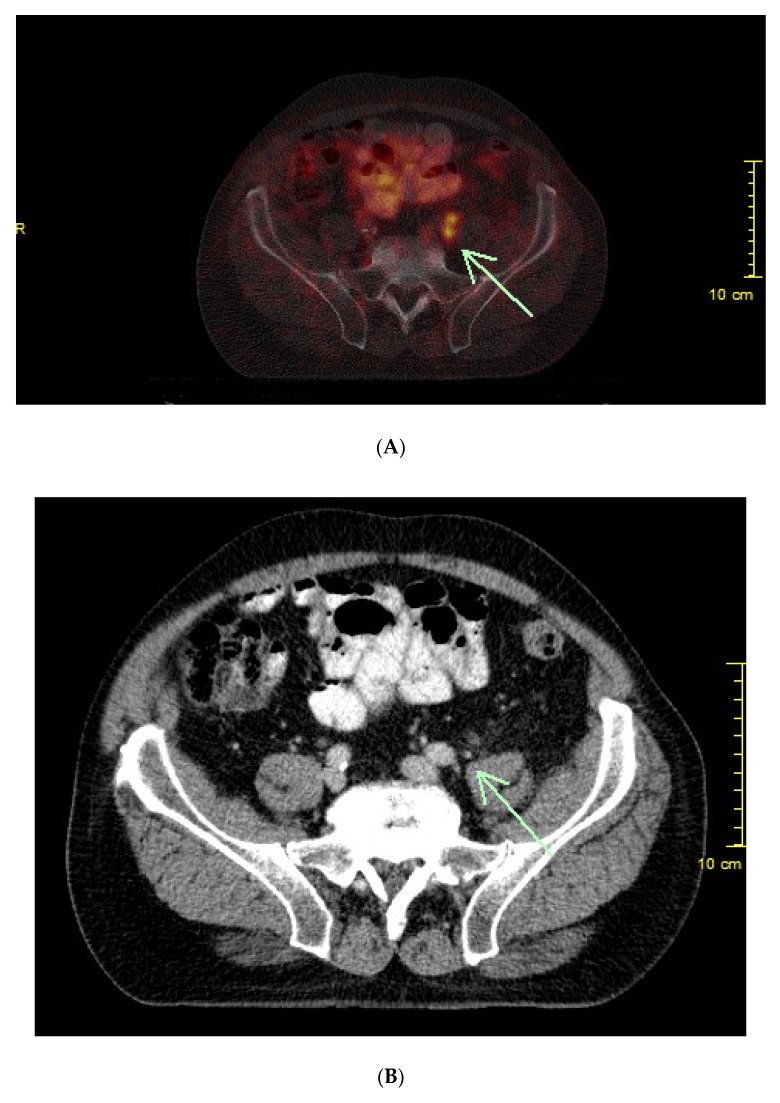
(**A**) History of a patient with prostate cancer followed by radical prostatectomy and lymphadenectomy (pT3, pN0, cM0, G3) in 2012. PSA doubles within three months. ^68^Ga-PSMA-11 PET/CT scan shows two lymph node metastases with high prostate specific membrane antigen (PSMA)-avidity. (**B**) The two small lymph node metastases are detectable on CT alone, but not suspect of malignancy.

We evaluated ^68^Ga-PSMA-11 PET/CT scans in 581 BC patients after primary curative PC therapy (RP as well as RT) in this retrospective study. A high positivity rate of 77% (450/581 patients) for the detection of malignant prostatic lesions was shown (Figure 4A,B). Our data reflects the positive results of several other studies and confirms that ^68^Ga-PSMA-11 PET/CT is a promising method for diagnosing PC [12,20,21,22,23,24]. For restaging prostatectomized BC patients by PSMA PET/CT, the EAU guidelines recommend a PSA level of > 0.2 ng/mL [8]. Patients with low cancer burdens have the best chance of a salvage RT to be curative. For this reason, imaging with subsequent therapy planning at an early stage of recurrent disease makes sense, from the point at which carcinoma foci are detectable, even with low PSA [25]. The EAU guidelines report that salvage RT in BC patients after RP was correlated with a tripling in PC-specific survival when compared to the patients who did not get salvage therapy [8]. The purpose of our study was to determine the best time for performing ^68^Ga-PSMA-11 PET/CT scans in BC patients. For that reason, we calculated the PSA and PSA kinetics thresholds for the detection and localization of recurrent PC. The PSA levels and PSA kinetics were assessed by ROC generated by plotting sensitivity versus 1-specificity.

### 3.1. Overall Positivity Rate

The positivity rate of ^68^Ga-PSMA-11 PET/CT of the present study was 77% over all patients (mean PSA 22.94 ± 115.38 ng/mL), thereby confirming the results of previous studies where detection rates of 74% to 83% were reported for restaging PET/CT [6,12,26]. There was a statistically significant ability to discriminate PSA values between PSMA-positive and PSMA-negative scans (*p* < 0.001). The overall patient group was divided into the group of patients who were primarily treated with prostatectomy (patient-group RP) and the group of patients who were primarily treated with RT (patient-group RT) to ensure clean statistics.

### 3.2. Positivity Rate (RP)

In the group of prostatectomized patients, the positivity rate was 75% (370/493 patients) (see Table 3A). Therefore, a clinically relevant percentage of patients with low PSA levels (< 0.5 ng/mL) could be diagnosed with the recurrence of PC, being comparable to the results of other studies [6,12,23,26]. In recent studies of restaging by ^68^Ga-PSMA PET/CT, the detection rate was 50% at PSA levels of 0.2 to 0.49 ng/mL [6] and, in another study, 57.9% of the patients were ^68^Ga-PSMA PET/CT-positive at PSA levels from 0.2 to < 0.5 ng/mL [20]. The results from the present study emphasize that ^68^Ga-PSMA PET/CT is a sensitive tool for restaging PC, even at low PSA values [23]. After early salvage RT (PSA ≤ 0.5 ng/mL), an undetectable PSA level can be reached in 60% of the patients, being associated with a chance of 80% for a progression free five-year survival. This is indicated as second line therapy with curative intent [8,25]. Salvage RT is usually indicated after a persistent increase in PSA after RP, when systemic metastases are not found during staging imaging. If conventional imaging does not show any malignant foci (local and/or distant metastases), only the prostate bed is typically irradiated by Radiation Oncologists. Eiber et al. described that the detection of malignant lesions by PET was extremely important for the final diagnosis in over 50%. They showed lesions that were not detected by computed tomography (CT) [20,23]. Another study demonstrated that, in patients with BC after RP while using the standard procedure, nearly 20% of ^68^Ga-PSMA PET/CT-positive lesions—suspicious of malignancy—would not have been included in the radiation field. All of these patients belonged to the crucial collective with PSA of < 1 ng/mL. ^68^Ga-PSMA-11 PET/CT hybrid imaging opens up the possibility of changing the therapy [23,27]. Based on the data, both radiation therapists and urologists should have a more accurate road map for directing therapy. In the end, the radiation therapist should point where to direct his device. If a LN is resectable, a urologist might also resect it.

In comparison to other radiopharmaceuticals (e. g. ^11^C- and ^18^F-choline), detection rates of ^68^Ga-PSMA at low PSA levels were higher [28,29]. PSA levels and ^11^C-choline scans with positive results were linearily correlated [30,31,32]. The positivity rate of ^11^C-choline scans for restaging was 73% at PSA levels of ≥ 3 ng/mL, but at PSA < 1 ng/mL the rate decreased dramatically to 19–36% [4,28]. However, a recent study showed that the second-generation of labelling of PSMA tracers with ^18^F (e.g., ^18^F-DCFPyL-PSMA) was superior to ^68^Ga-PSMA for restaging at PSA levels of 0.5 to 3.5 µg/L with a sensitivity of 88% (15 of 17 patients) vs. 66% (23 of 35 patients). But at PSA values < 0.5 and > 3.5 µg/L, the sensitivity of both methods was comparable. Nevertheless, in 36% of the patients with positive scans, additional lesions were found, while using ^18^F-PSMA vs. ^68^Ga-PSMA [18]. Overall, the results of both methods were demonstrated to be similar [18]; this was also shown in a recent study, which compared ^68^Ga-PSMA-11 and ^18^F-PSMA-1007 in the case of staging PC [33].

### 3.3. Positivity Rate (RT)

The positivity rate of RT patients was 91%, but no statistically significant differences could be shown in the distinction of PSA levels between patients with positive and negative scans (*p* = 0.384) (see Table 3B). The EAU Guidelines recommend that PSMA PET/CT can be crucial for PC detection in the setting of BC after RT. However, they suggest that further studies be conducted [8].

### 3.4. PSA and PSA Kinetics Threshold Levels

We assessed the performance of ^68^Ga-PSMA-11 PET/CT regarding pre-scan PSA levels by ROC curve separating the two patient-groups RP and RT. Based on the results of previous studies [4,9], which emphasized the importance of PSA kinetics for patient selection and detection, we also calculated PSAvel and PSAdt (see introduction), as they are valuable parameters for the prediction of clinical progression. However, it should also be borne in mind that there are non-PSA-producing tumor masses in metastatic and fully treated PCs.

### 3.5. PSA and PSA Kinetics Threshold Levels (RP)

In our study, a PSA of 1.24 ng/mL was the optimal cutoff level for distinguishing between positive and negative ^68^Ga-PSMA-11 PET/CT scans. ^68^Ga-PSMA-11 PET/CT showed the presence of multimetastatic PC in 21% of the positive lesions in patients with PSA levels below the cutoff value and in 79% in patients with PSA above the cutoff (Table 5A). Thus, we conclude that the cutoff by hybrid imaging with ^68^Ga-PSMA-11 can be used for patient selection for restaging and also fits well into clinical practice. If you convert 1.24 ng/mL to tumor mass, it might be a small LNM or tissue volume of less than one cubic milliliter.

Significant differences between PET-positive and PET-negative patients were calculated with corresponding optimal PSAvel threshold of 1.32 ng/mL/year (*p* < 0.001). Similarly to other studies, our data show higher positivity rates corresponding to a higher PSAvel, which was also seen in studies with ^18^F-choline. Like other authors, we propose that in addition to the PSA level, PSAvel could be taken into account in patient selection [9,20,32,34]. On the other hand, a recent study, which assessed a large cohort of patients, did not show significant correlation between ^68^Ga-PSMA-11 PET/CT positivity and PSAvel [35].

In this study, PSAdt was not significantly associated with ^68^Ga-PSMA-11 PET/CT positivity. For distinguishing between the positive and negative PSMA-PET findings, no optimal PSAdt cutoff could be determined by ROC analysis (AUC = 0.450). The value of PSA in patient selection is controversial [9]. Verburg et al. recommended combining serum PSA with PSAdt in order to achieve the best PSMA-positive scan result or the detection of distant LNM; in which they emphasized the importance of PSA over that of PSAdt [26]. Ceci et al. demonstrated PSAdt as a valuable predictor of positive ^68^Ga-PSMA PET/CT scans and pointed out that patients with a short PSAdt and low PSA values showed positive PSMA scans in 85% [36]. Corresponding to the EAU guidelines, these patients were ideal candidates for salvage RT, as salvage RT has been described to be mostly efficient in patients with short PSAdt [8,36]. In contrast, in the same study, 18.7% of patients had low PSA values, but long PSAdt, and also showed positive PSMA-PET/CT findings [36].

### 3.6. PSA and PSA Kinetics Threshold Levels (RT)

In our study, we calculated an optimal PSA cutoff level from ROC curve of 5.75 ng/mL (Table 5B). We were not able to determine an optimal PSAvel threshold for the irradiated patient-group based on the available data. ROC analysis revealed a PSAdt cutoff of 10.6 months for the identification of RT patients with positive and negative scans, which did not find significance (*p* = 0.353).

At the present time, our cutoff results cannot be compared with the results of other studies in terms of thresholds, since they consider both (the prostatectomized and the irradiated patients) in one common patient group. Otherwise, only the RP-group is analyzed. Radiation groups are rarely examined alone for calculating cutoff levels.

### 3.7. Subpopulations

The performance of ^68^Ga-PSMA-11 PET/CT has to be appraised for a therapeutic approach. In particular, the detection of local LNM should be considered, which could lead to a changed therapeutic approach (salvage RP plus LNM dissection or salvage RT with extended radiation field). We took a closer look at PSA range between 0.2 ng/mL to < 1 ng/mL for post-prostatectomized patients based on the EAU guidelines for performing PSMA PET/CT in different initial situations [8]. The guidelines do not specify a threshold after primary radiation, so we have not formed a subgroup for these patients [8]. In the RP-group, 29% of patients showed PSA values that were between 0.2 and < 1 ng/mL with a PSMA-positivity rate of 52%. There was a significant association between the pre-scan PSA and an increased likelihood of a positive PSMA-scan was proven (*p* < 0.001). In comparison, Eiber et al. demonstrated positive scans in 57.9% for PSA values of 0.2 to < 0.5 ng/mL and even 72.7% at 0.5 to < 1 ng/mL [20]. A published study by Graziani et al. showed that only about 45% of the scans were positive while using hybrid imaging with ^11^C-choline PET/CT in patients, even with PSA levels between 1 and 2 ng/mL. Multimetastatic disease was detected in 38% of all scans and distant lesions in 19% of the patients with PSA levels between 1–2 ng/mL [37]. In the present study, ^68^Ga-PSMA-11 PET/CT revealed local metastases in 59%, multimetastatic disease in 44%, and distant lesions were shown in 13% of the subpopulation patients. A clinically relevant number of patients can be selected for salvage therapy due to the effectiveness of ^68^Ga-PSMA-11 PET/CT for the detection of metastases, which is apparently higher than that of ^11^C-choline, possibly even for metastatic-directed treatment, which might justify salvage LN dissection rather than systemic therapeutic path, thereby help to avoid unnecessary procedures and complications and also improve the chance of a clinical recurrence-free survival [38,39].

### 3.8. GS

Histopathological GS was significantly associated with positive ^68^Ga-PSMA-11 PET/CT results (*p* < 0.001). Patients with GS of 7b (intermediate risk and grade group 3) and > 7 (high risk and grade group 4 or 5) showed an increased incidence of positive ^68^Ga-PSMA-11 PET/CT scans (*p* < 0.001). Between patients with PSMA-positive scans corresponding to GS 7a (intermediate risk and grade group 2) and PSMA-positive scans corresponding to GS 7b, there was a significant demarcation (*p* < 0.001). Regarding the clinically important, highly significant differentiation between GS 7a (3 + 4) and 7b (4 + 3), patients with GS 7b exhibited more frequent pathologic ^68^Ga-PSMA-11 PET/CT scans and metastases [8,40]. Therefore, the findings of hybrid imaging with ^68^Ga-PSMA-11 agree in many cases with higher risk relapses on the basis of histology (frequency and detection of metastases). Our results are in line with other studies, which could possibly be due to the fact that the PSMA expression is usually more intense in higher-grade GS lesions than in lesions with a lower-grade GS [8,12,23,41]. Our results correspond to the immunohistochemical results of PSMA expression in PC [20].

### 3.9. ADT

The positivity rate of ^68^Ga-PSMA-11 PET/CT was significantly correlated with accompanying ADT in this study. This is in line with the results of a study, in which the ADT-treated patients revealed positive PET/CT results more often and that was believed to be due to the frequent use of ADT in patients with advanced disease [20]. The same phenomenon has also been described when using ^11^C-choline [37]. Furthermore, there have been reports of higher PSMA-expression of PC tumor cells as part of ADT [42]. Overall, the scientific knowledge on this issue remains unclear and further studies are needed [12,20].

### 3.10. AP

In our study, patients with PSMA-positive lesions showed higher AP levels than patients with negative PSMA scans (*p* < 0.001). Additionally, the detection of bone metastases was significantly associated with high AP. Our results corroborate the findings of other studies where serum AP was found to be a predictor for bone involvement of PC and to be an efficient and reliable biomarker for the prognosis of PC [43]. The AP levels were higher in patients with positive ^68^Ga-PSMA-11 PET/CT scan results, with higher PSA levels, in patients receiving ADT and in patients exhibiting bone metastases in our study. Li et al. suggest that high AP is significantly related to poor overall survival and poor progression-free survival in PC, but there is no apparent association to cancer specific survival [43]. Skeletal AP is prognostic for bone metastases for M-staging of PC, and it is a dependable marker of osteoblastic activity. Another study demonstrated that AP levels were more closely associated with pathological bone scans than with PSA values and that the amount of AP was positively related to bone metastases [44]. From this, we conclude that the term “bone-specific AP” can reasonably be used.

## 4. Materials and Methods

### 4.1. Patient Characteristics

581 BC patients that had previously been treated with RP or RT, underwent ^68^Ga-PSMA-11 (Glu-urea-Lys(Ahx)-HBED-CC) PET/CT between 2015 and 2019 and were retrospectively evaluated. The inclusion criteria for the performance of a ^68^Ga-PSMA-11 PET/CT scan were: (a) histopathologically proven primary PC; (b) treatment with RP or RT (with or without ADT); and, (c) proven BC by definition (BC is defined as an elevation of the PSA value after primary treatment to > 0.2 ng/mL after RP or > 2 ng/mL above nadir following RT). The data on some of the patients included were already considered in another study with different aim. The PSA levels were available at the time of surgery as well as nadir, interim, and PET/CT scans (BC). Mean relapse PSA levels were 18.21 ng/mL ± 101.91 ng/mL (median 2.98, range 0.2–2000). 209 patients were treated with anti-androgens (Table 1). The patient data were collected from four Nuclear Medicine Institutions (a–d) in Germany. We have classified the patients into the following groups: (1) To obtain a clean statistic, we looked at the patient-group after RP and the patient group after RT separately, as, by definition, they have a different BC. (2) The subgroup of RP-patients was divided into five groups: 0.2 to < 0.5 ng/mL, 0.5 to < 1 ng/mL, 1 to < 2 ng/mL, 2 to < 5 ng/mL, and 5 ng/mL, and above. (3) The RT-patients were divided into two groups: 2 to < 5 ng/mL and 5 ng/mL and above. Additionally, patients were assigned to five-tiered GS groups: < 7, 7a and 7b, 8 and > 8, respectively. The following parameters were evaluated: ^68^Ga-PSMA PET/CT scan results, PSA values, PSA kinetics, SUV_max_ of the malignant lesions, GS, ADT, AP and local/distant/single/multiple metastases, regional and/or distant LNM, bone metastases, and visceral metastases (e.g., lung). The main limitation of this retrospective study as compared to many other retrospective studies is that most of the patients did not have histopathological confirmation of the lesions. As a result, we use the term positivity rate instead of detection rate in this study. In addition to the histopathological findings (when available), we used a significant increase in PSA or a decrease in PSA after subsequent therapy (e.g. salvage RT) or a confirmation of the pathological focus in follow-up imaging or an increase and/or enlargement of the lesions in follow-up imaging, as indicators. We evaluated the optimal cutoff level by calculating PSA and PSA kinetics thresholds while using the ROC curve to determine the best time to perform ^68^Ga-PSMA-11 PET/CT scans in patients with BC. The present study was in accordance with the Helsinki Declaration and the German Medicinal Products Act, AMG §13.2b. The retrospective study was conducted in accordance with the Declaration of Helsinki, and the Ethics Committee of Laek Rlp (2018-13390, approval date: 29 October 2018) and Aek No (41/2019, approval date: 22 February 2019) approved the protocol. All of the patients gave their written informed consent for the examination and for inclusion in the study.

### 4.2. Radiopharmaceuticals

The ^68^Ga-labelled PSMA ligand, Glu-urea-Lys(Ahx)-HBED-CC (^68^Ga-PSMA-11), was synthesized while using modification of a method described in 2012 [16]. In short, ^68^Ga was achieved from a ^68^Ge/^68^Ga radionuclide generator (Garching, Germany) to be used to label the PSMA ligand. The labelled tracer without carrier additive was cleaned using a reverse phase cartridge (Sep-Pak C18 Plus Light cartridge, 130 mg Sorbent; Waters) and formulated in 10 mL phosphate-buffered saline with 5% by volume ETOH. Confirmation by radio thin-layer chromatography (TLC) and high-performance liquid chromatography (HPLC) was able to show a radiochemical purification yield of over 98%. ^68^Ga-PSMA-11 was obtained from the Clinic of Nuclear Medicine of the Medical Center of the University Johannes Gutenberg (Mainz, Germany; b, c), from Advanced Accelerator Applications (Bonn, Germany; b, c), from Eckert & Ziegler (Bonn, Germany; d) or was produced in house (Mainz, Germany; a), depending on the availability.

### 4.3. Imaging Protocol

Each patient received an intravenous injection of ^68^Ga-PSMA-11 (mean and SD: 199 ± 61 MBq, median activity: 200 MBq, range: 50–390 MBq). The PET data of tracer distribution were acquired 60 ± 10 min. (whole body) after injection of ^68^Ga-PSMA-11. The patients were imaged on a PET/CT scanner named Gemini TF16 (Philips Medical Systems, Best, The Netherlands) (a, c) or a PET/CT scanner, named Biograph 64/Z64, R4 (HD and time of flight)/Biograph 64 TruePoint (True V HD; Siemens, Erlangen, Germany) (b, d). Either a mid-inspiratory low-dose CT scan (120 kV, 20–60 mAs, CT transverse scan field 50 cm, 70 cm extended field of view, high contrast resolution 1.0 s, 0.6 mm) without contrast enhancement or a maximum inspiratory venous-phase diagnostic contrast enhanced (Ultravist 300; Bayer AG, Berlin, Germany) CT scan (140 kV, 100–400 mAs, dose modulation) from the head to the upper-thigh was used for anatomical correlation and for attenuation corrections. PET was performed while using a standard technique on a dedicated three-dimensional (3D) system (a, c: matrix 144 × 144; d: 400 × 400; b: 168 × 168), with an acquisition time of 3 min. per bed position (axial field of view d: 13.3 cm; b: 21.8 cm; c: 19 cm; a: 18 cm). Random, scatter, and decay correction were applied to the emission data. Ordered-subsets expectation maximization method (OSEM) (a: three iterations, 33 subsets, Gaussian filtering, 4.3 mm full-width at half-maximum; b, c: two iterations, 14 subsets, Gaussian filtering, 5 mm (c) or 4.2 mm (b) transaxial resolution, full-width at half-maximum; d: three iterations, 24 subsets, Gaussian filtering, 5 mm transaxial resolution, full-width at half-maximum) was used for PET image reconstruction. The CT data were converted to attenuation coefficients at 511 keV and applied for attenuation corrections of the PET images.

### 4.4. Imaging Analysis

The PET/CT images were first visually assessed by individual specialists (nuclear medicine physician, radiologist) while applying coronal, transaxial, and sagittal layers. Subsequently, all of the images were interpreted in consensus by at least one experienced nuclear medicine physician, and two experienced radiologists, each of them board-certified and with PET/CT experience of more than five years as well as rich experience in interpretation of hybrid imaging with ^68^Ga-PSMA-11. The final diagnosis was reached by consensus. Any PSMA-avid lesion with a morphological substrate on CT and an uptake above the background (but not correlated with physiological tracer uptake) was considered as being suggestive of PC. The SUV_max_ was measured for all lesions, suspicious of malignancy. PSMA-PET-avid lesions as described above were classified as suspected local recurrence in the prostate or prostate bed, as local/regional LN in the pelvis (iliac and/or pararectal) or as distant/extrapelvic LN (retroperitoneal and/or above the iliac bifurcation), as well as bone metastases and visceral metastases (lung, liver, adrenals, soft tissue, spleen, thyroid). When conflicting interpretations were identified, they were discussed and solved by the panel and consensus was passed between the reviewing physicians.

### 4.5. Statistical Analysis

Statistical analyses were performed while using SPSS version 24.0 (IBM Corporation, Ehningen, Germany). For continuous metric normally distributed variables, Students t-test was performed to determine differences between two groups. Multivariate variance analysis was used for comparison of more than two groups. We analyzed non-normally distributed continuous variables by Mann-Whitney-U test. The Wilcoxon signed rank test was calculated for determining the changes of PSA levels. For further analysis, we subdivided the metric values (e.g., PSAvel and PSAdt) into categories. We analyzed these nominal and ordinal parameters with Chi-square test and Pearson correlation. The performance of ^68^Ga-PSMA-11 PET/CT was assessed in relation to these parameters by ROC curves in order to determine the optimal cutoff of PSA levels and of PSA kinetics to differentiate between positive and negative scan results. The data are presented as mean, SD, and/or median and range. *p* < 0.05 was considered to be significant.

## 5. Conclusions

This study identifies the PSA and PSA kinetics thresholds for the presence of ^68^Ga-PSMA-11 PET/CT-detectable PC-lesions in patients with BC. Pre-scan PSA was the main predictor of a positive scan with an optimal cutoff level of 1.24 ng/mL for patients that were primarily treated with RP. In this subgroup, kinetics analysis of PSA calculated a threshold velocity of 1.32 ng/mL/year.

We conclude that the PSA levels and kinetics (PSAvel) are suitable risk markers for optimizing the selection of patients who may benefit from ^68^Ga-PSMA-11 PET/CT, especially in the subgroup of prostatectomized patients. ^68^Ga-PSMA-11 PET/CT has a great impact on selecting patients with primary RP for salvage RT with curative intent.

## Figures and Tables

**Table 1:** Patient characteristics.

Characteristics (*n*)	Parameters
Number of patients	581
Age (y) (581)	
Median	71
Range	49–88
Mean ± SD	71.3 ± 7.5
Gleason Score (581)	
≤ 6 (low risk + grade group 1)	37
7 (intermediate risk + grade group 2+3)	303
8 (high risk + grade group 4)	104
> 8 (high risk + grade group 5)	139
PSA (ng/mL) (581)	
Median	2.98
Range	0.2–2000
Mean ± SD	18.21 ± 101.91
PSAvel (ng/mL/y) (196)	
Median	1.24
Range	0–620.1
Mean ± SD	10.89 ± 54.25
PSAdt (months) (581)	
Median	10.35
Range	0–628.2
Mean ± SD	22.1 ± 48.8
Prior treatment of primary tumor (581)	
Surgery (radical prostatectomy)	493
Radiotherapy and other	88
Further treatment	
Anti-androgen therapy	209
Positivity rate	
Total/PET/CT positive patients	450/581

Abbreviations: PSA, prostate-specific-antigen; vel, velocity; dt, doubling time; SD, standard deviation; n, number of patients; y, year.

**Table 2:** ^68^Ga-PSMA-11 positron-emission tomography/computed tomography (PET/CT)-11 positive and negative scan results in relation to prostate-specific-antigen (PSA).

PET/CT Results	PSA (ng/mL)		
	Mean ± SD	Median (range)	*p* Value
**Positive (450/581)**	22.94 ± 115.38	4.01 (0.2-2000)	
**Negative (131/581)**	1.95 ± 3.28	0.8 (0.2–25.67)	
**Total**	18.21 ± 101.91	2.98 (0.2–2000)	*p* < 0.001 *

* Mann-Whitney-U test. Abbreviations: PSA, prostate-specific-antigen; SD, standard deviation; *p* < 0.05 is considered significant.

**Table 3:** (**A**) Prostate cancer (PC) recurrence (patient-group radical prostatectomy (RP)) location related to different PSA values. (**B**) PC recurrence (patient-group RT) location related to different PSA values.

**(A) Prostate cancer (PC) recurrence (patient-group radical prostatectomy (RP)) location related to different PSA values.**						
**PSA (ng/mL)**	**0.2–<0.5**	**0.5–<1.0**	**1.0–<2.0**	**2.0–<5.0**	**≥5.0**	**Chi², *p***
Number (x/493) patient-group RP	67	78	87	118	143	
PET/CT positive	27	48	61	99	135	*r* = 0.413; *p* < 0.001
Positivity rate	40.3%	61.5%	70.1%	83.9%	94.4%	
Androgen deprivation therapy	15	13	21	31	77	*r* = 0.252; *p* < 0.001
Regions:						
Local recurrence	6	14	19	29	41	*r* = 0.149; *p* = 0.02
Metastases	22	39	49	80	123	*r* = 0.365; *p* < 0.001
Site of metastases:						*r* = 0.402; *p* < 0.001
Local metastases	16	28	32	43	50	
Distant metastases	3	7	12	18	31	
Local + distant metastases	3	4	5	19	42	
Number of metastases:						*r* = 0.397; *p* < 0.001
Single metastases	14	14	23	25	29	
Multiple metastases	8	25	26	55	94	
Lymph node metastases (LNM)	19	30	38	61	97	*r* = 0.266; *p* < 0.001
Site of LNM:						*r* = 0.344; *p* < 0.001
Local LNM	18	28	34	51	52	
Distant LNM	0	0	2	4	13	
Local + distant LNM	1	2	2	6	32	
Bone metastases	8	11	12	33	72	*r* = 0.315; *p* < 0.001
Visceral metastases	1	1	2	5	12	*r* = 0.128; *p* = 0.075 *
**(B) PC recurrence (patient-group RT) location related to different PSA values.**						
**PSA (ng/mL)**	**2.0–<5.0**		**≥5.0**		**Chi², *p***	
Number (x/88) patient-group RT	33		55			
PET/CT positive	29		51		*r* = 0.08; *p* = 0.44	
Positivity rate	87.9%		92.7%			
Androgen deprivation therapy	11		41		*r* = 0.406; *p* = 0.001	
Regions:						
Local recurrence	21		29		*r* = −0.107; *p* = 0.317	
Metastases	14		37		*r* = 0.244; *p* = 0.022	
Site of metastases:					*r* = 0.306; *p* = 0.011	
Local metastases	10		12			
Distant metastases	1		13			
Local + distant metastases	3		12			
Number of metastases:					*r* = 0.289; *p* = 0.022	
Single metastases	6		7			
Multiple metastases	8		30			
Lymph node metastases (LNM)	12		23		*r* = 0.054; n.s.	
Site of LNM:					*r* = 0.076; n.s.	
Local LNM	9		17			
Distant LNM	2		1			
Local + distant LNM	1		5			
Bone metastases	5		22		*r* = −0.261; *p* = 0.014	
Visceral metastases	2		1		*r* = −0.113; *n.s*.	

* Fisher exact test. Abbreviations: PSA, prostate-specific-antigen; LNM, lymph node metastases; *p* < 0.05 is considered significant; r, Pearson correlation coefficient.

**Table 4:** Tumor location and positivity rate of detected lesions.

Tumor Location	*n* (493)	% (370)	*n* (88)	% (80)
PET/CT positive patients	370		80	
Local recurrence	109	29.5%	50	62.5%
Metastases	313	84.6%	51	63.8%
local	169	45.7%	22	27.5%
distant	71	19.2%	14	17.5%
local and distant	73	19.7%	15	18.8%
single	105	28.4%	13	16.3%
multiple	208	56.2%	38	47.5%
Lymph node metastases	245	66.2%	35	43.8%
Local/regional	183	49.6%	26	32.5%
Distant	19	5.1%	3	3.8%
Local and distant	43	11.6%	6	7.5%
Bone metastases	136	36.8%	27	33.8%
Visceral metastases (lung, liver, adrenals, soft tissue, spleen, thyroid)	21	5.7%	3	7.5%

Abbreviation: *n*, number of patients.

**Table 5:** (**A**) ^68^Ga-PSMA-11 PET/CT positivity rate (RP) of different subgroups related to PSA levels. (**B**) 68Ga-PSMA-11 PET/CT positivity rate (RT) of different subgroups that were related to PSA levels.

**(A) ^68^Ga-PSMA-11 PET/CT positivity rate (RP) of different subgroups related to PSA levels.**						
**PSA Range (ng/mL)**	**Overall Positivity**	***p*/*r* Value**	**Single Metastases**	**Multiple Metastases**		***p*/*r* Value**
0.2 to <1 (145)	75 (51.7%)		28 (19.3%)	33 (22.8%)		
<1.24 (177)	92 (52%)		33 (31%)	44 (21%)		
≥1.24 (316)	278 (87.4%)		72 (69%)	164 (79%)		
Total (493)	370 (75%)	*p* < 0.001: *r* 0.400	105	208		*p* < 0.001: *r* 0.315
**PSA Range (ng/mL)**	**Local Recur-Rence**	***p*/*r* Value**	**Local Metastases**	**Distant Metastases**	**Local + Distant Metastases**	***p*/*r* Value**
0.2 to <1 (145)	20 (13.8%)		44 (30.3%)	10 (6.9%)	7 (4.8%)	
<1.24 (177)	23 (13%)		51 (28.8%)	15 (8.5%)	11 (6.2%)	
≥1.24 (316)	86 (27.2%)		118 (37.3%)	56 (17.7%)	62 (19.6%)	
Total (493)	109	*p* < 0.001; *r* 0.164	169	71	73	*p* < 0.001; *r* 0.308
(**B**) **^68^Ga-PSMA-11 PET/CT positivity rate (RT) of different subgroups that were related to PSA levels.**						
**PSA Range (ng/mL)**	**Overall Positivity**	***p*/*r* Value**	**Single Metastases**	**Multiple Metastases**		***p*/*r* Value**
<5.75 (36)	31 (86.1%)		15 (41.7%)	19 (52.8%)		
≥5.75 (52)	49 (94.2%)		7 (13.5%)	29 (55.8%)		
Total (88)	80 (91%)	*p* = 0.19; *r* 0.139	22	48		*p* = 0.008; *r* 0.255
**PSA Range (ng/mL)**	**Local Recur-Rence**	***p*/*r* Value**	**Local Metastases**	**Distant Metastases**	**Local + Distant Metastases**	***p*/*r* Value**
<5.75 (36)	22 (61.1%)		11 (30.6%)	1 (2.8%)	4 (11.1%)	
≥5.75 (52)	28 (53.8%)		11 (21.2%)	13 (25%)	11 (21.2%)	
Total (88)	50	*p* = 0.5; *r* −0.07	22	14	15	*p* = 0.01; *r* 0.286

Abbreviations: PSA, prostate-specific-antigen; *p* < 0.05 is considered significant; *r*, Pearson correlation coefficient.

**Table 6:** Gleason Score in relation to ^68^Ga-PSMA-11 PET/CT-11 positive scan results.

*n* = 581	GS < 7 (37)	GS 7a (97)	GS 7b (204)	GS 8(104)	GS > 8(139)	Chi^2 ^*r*, *p* Value
**PET/CT positive (450/581)**	16	52	158	97	117	0.288; *p* < 0.001
**Local recurrence (159/581)**	9	24	56	32	38	0.027; n.s.
**Metastases (364/581)**	8	36	132	83	105	0.324; *p* < 0.001

Abbreviations: GS, Gleason Score; *n*, number of patients; *p* < 0.05 is considered significant; *r*, Pearson correlation coefficient.
